# Contrast‐enhanced transcranial Doppler for the detection of right‐to‐left shunt: A new provocation method with a syringe‐modified Valsalva maneuver

**DOI:** 10.1002/brb3.3304

**Published:** 2024-05-17

**Authors:** Jinfeng Deng, Yan Luo, Shijian Luo, Hongrui Zhan, Feng Zhou, Songbiao Li

**Affiliations:** ^1^ Department of Neurology The Fifth Affiliated Hospital of Sun Yat‐sen University Zhuhai China; ^2^ Department of Rehabilitation The Fifth Affiliated Hospital of Sun Yat‐sen University Zhuhai China; ^3^ Cardiovascular Center The Fifth Affiliated Hospital of Sun Yat‐sen University Zhuhai China

**Keywords:** contrast‐enhanced transcranial doppler, detection rate, right‐to‐left shunt, Valsalva maneuver

## Abstract

**Background:**

Contrast‐enhanced transcranial Doppler (cTCD) study has been established as one of the most common investigations for detecting right‐to‐left shunt (RLS). Although the conventional Valsalva maneuver (c‐VM) has been used to increase the sensitivity of cTCD for RLS, efforts are still needed to improve the detection rate further. We proposed a new provocation method with a syringe‐modified Valsalva maneuver (sm‐VM) during cTCD and compared the efficacy of this strategy with cTCD measured at resting and with the provocation of c‐VM.

**Methods:**

Consecutive patients with suspicion of RLS who underwent cTCD in our institution between September 27, 2021, and April 1, 2022, were included in this study. Examination of cTCD was performed separately at the resting state and provoked with c‐VM and sm‐VM. The overall proportion of patients with RLS and their distribution with different RLS grades were compared.

**Results:**

A total of 389 patients (mean age: 49.37 years, male: 52.2%) were included in this study. The positive rate for RLS was significantly higher for cTCD detected with sm‐VM than those detected at resting state and with c‐VM (46.8% vs. 21.6% and 34.2%, all *p* < .05). Besides, cTCD detected with sm‐VM was also associated with a higher proportion of patients with grade III RLS than those detected at resting state and with c‐VM (11.3% vs. 1.8% and 0%, all *p* < .05).

**Conclusions:**

Compared to cTCD detected at resting state and with c‐VM, cTCD with sm‐VM could further increase the positive detection rate of RLS.

## Graphical abstract

A new provocation method with a syringe‐modified Valsalva maneuver (sm‐VM) during Contrast‐enhanced transcranial Doppler (cTCD) was proposed in this study. Compared to cTCD detected at resting state and with conventional Valsalva maneuver, cTCD with sm‐VM could further increase the positive detection rate of RLS. These findings support the use of this method in clinical practice.

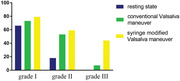

## 1 INTRODUCTION

Right‐to‐left shunt (RLS) is an abnormal circulatory disorder that could be classified as intracardiac shunt and extracardiac shunt based on the shunt location (Burkett, 2020; Driscoll, 1999). Intracardiac RLS is clinically caused by congenital heart diseases, including the patent foramen ovale (PFO), atrial septal defect, and ventricular septal defect. Extracardiac RLS is mainly caused by defects of the patent ductus arteriosus and pulmonary arteriovenous fistula (Vijayalakshmi, 2015; Zanchetta et al., 2005). PFO is the predominant cause of RLS, accounting for approximately 95% of patients with RLS (Mazzucco et al., 2018). Accumulating evidence suggests that RLS has been related to the pathogenesis of embolic strokes of undetermined source (Takaya et al., 2021), migraine (Cao et al., 2022), and decompression illness (Wilmshurst, 2015), which adversely affect the quality of life and health of the global population. Previous studies showed that the prevalence of PFO in some adult populations varied between 20% and 30% (Hagen et al., 1984; Kuramoto et al., 2015; Meissner et al., 2006). Many people with PFO in the global population and the possible adverse influence of RLS highlight the importance of RLS detection in a high‐risk population for preventing and treating RLS‐related clinical syndromes.

Current diagnosis of RLS in clinical practice depends on a comprehensive evaluation of multidimensional information, including the clinical manifestation of the patients and the findings of a variety of imaging examinations, including contrast transthoracic echocardiography (cTTE), contrast transesophageal echocardiography (cTEE), contrast‐enhanced transcranial Doppler (cTCD), and intracardiac echocardiography (ICE) (Katsanos et al., 2016; Messe et al., 2020; Zetola et al., 2019). Among these imaging investigations, cTTE, cTEE, and ICE are images based on two‐dimensional tomography. These examinations based on a single image may miss the signs of RLS in the left atrium, which causes the missed diagnosis (Katsanos et al., 2016). Besides, as an invasive examination, cTEE may cause discomfort during the examination and may not be well accepted by all patients in clinical practice (Maillet et al., 2018). Compared to these imaging investigations, cTCD is noninvasive and the only real‐time monitoring method for detecting microembolisms passing through the cerebrovascular arteries to reflect the existence of RLS (Mojadidi et al., 2014). Besides, the findings of cTCD in patients with RLS may be more closely related to the potential neurological symptoms of the patients, thereby conferring significant therapeutic implications (Messe et al., 2020). Therefore, cTCD has been recommended as one of the most commonly used imaging examinations for screening RLS in real‐world clinical practice.

Previous studies have shown that compared to cTCD examination under a resting state, cTCD provoked by the conventional Valsalva maneuver (c‐VM) may further increase RLS's detection rate and the sensitivity of the examination (Thiagaraj et al., 2019). Accordingly, cTCD examinations for RLS are generally performed both under the resting state and with the provocation of c‐VM (Van der Giessen et al., 2020). However, the performance of c‐VM lacks intuitive and quantitative performance indicators. Some patients have difficulty understanding and effectively performing the c‐VM during the cTCD examinations, limited by educational levels and comorbidities (He et al., 2020). All of these factors may cause the missed diagnosis of RLS in these patients. To overcome the above limitations, Dr. Spencer first described the manometer use for cTCD bubble study about 20‐year ago (Spencer et al., 2004). Each patient was instructed to blow into a mouthpiece attached to a manometer until 40 mmHg pressure was achieved and maintained for 10 s, as the calibrated Valsalva maneuver (Spencer et al., 2004). This process is easy to understand and conduct and could provide a quantitative effect of the Valsalva maneuver. However, because a manometer is still needed, the use of the process may also be limited. Here, we proposed a new provocation method with a syringe‐modified Valsalva maneuver (sm‐VM) during cTCD and compared the efficacy of this strategy with cTCD measured at resting and with the provocation of c‐VM. This sm‐VM‐provoked cTCD examination may become a practical and effective strategy for detecting RLS in high‐risk patients.

## 2 METHODS

This study was approved by the Research Ethics Committee of The Fifth Affiliated Hospital of Sun Yat‐sen University (approval series number 2022[L010‐1]) before the performance of the study. All of the included patients provided written consent before enrollment.

### 2.1 Patients

Consecutive patients suspected of RLS who underwent cTCD in the Department of Neurology, the Fifth Affiliated Hospital of Sun Yat‐sen University, between September 27, 2021 and April 1, 2022, were included in this study. Patients who fulfilled all of the following criteria were included in the study: (1) aged between 18 and 75 years; (2) fully acknowledged the procedure of the study, volunteered to participate in the study, and were able to cooperate during the procedure; and (3) with clinical suspicion of RLS as suggested by symptoms, including dizziness, headache, indistinct speech, limb weakness or numbness, blurred vision, and transient unconsciousness. In our institution, the Cardiology Department also undertakes the screening task of cerebrovascular disease for some time. In the process of cardiovascular clinical practice based on our experience, it was found that for some patients with asymptomatic mild tricuspid valve regurgitation, mild left atrial enlargement, mild pulmonary hypertension, hypertension, or diabetes, TCD screening may be an examination with different significance from ordinary Doppler examination. So, some asymptomatic patients were included in the study population. Patients who had either of the following clinical conditions were excluded: (1) patients with insufficient penetration of temporal window; (2) patients with aortic valve stenosis; (3) patients who were diagnosed with myocardial infarction, glaucoma, retinopathy, pregnancy, and foramen ovale occlusion within 1 month; (4) those who have mental and psychological diseases which were believed to be unable to fully cooperate during the study procedure; (5) patients with local skin infection; (6) patients with speech impairment; and (7) patients with grade II‐IV pulmonary function.

### 2.2 Procedures of cTCD examinations

For all of the included patients, the cTCD examination was performed via the DWL transcranial Doppler instrument (DWL Doppler‐BOX) with QL modular software (embolic detection and monitoring) by the same experienced physician. The patient was kept supine and examined with the single channel double depth mode after opening the embolic monitoring software. The probe was placed in the temple on the same side to detect the middle cerebral artery's (MCA) embolic signal on one side. The right antecubital vein was catheterized and then connected with a three‐way connector. The three‐way connector was respectively connected with two 10 mL syringes. The first one contains 8 mL of normal saline and 1 mL of blood from the patient himself. The second syringe contained 1 mL of clean air, which was then injected and mixed with the normal saline and blood back and forth by hand to prepare the activated normal saline of mixed blood (medium). The 10 mL medium bolus was then injected by a study nurse. From the beginning of the injection, the microbubbles (MBs) were monitored and recorded within 20 s by the physician during the cTCD examination. Then, the examination on the other side was repeated similarly.

**FIGURE 1:**
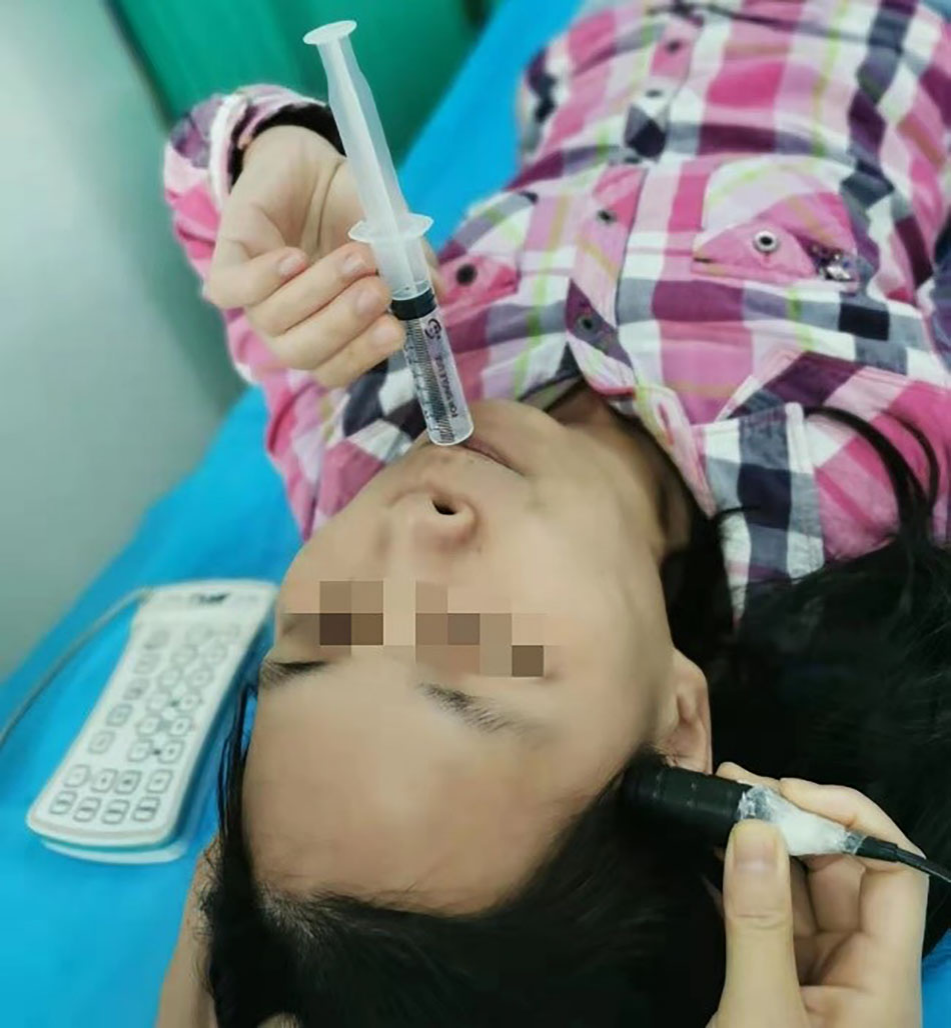
Representative image showing the process of contrast‐enhanced transcranial Doppler (cTCD) examination provoked with a syringe‐modified Valsalva maneuver. The photo is a simulation by the first author herself, mainly to explain the implementation process of the patient's end‐blowing movement.

For each patient, the cTCD examination was performed under three conditions, respectively: (1) a resting state, in which the patient was asked to lie still for at least 5 min without any VM; (2) a provoked state with c‐VM, in which the patient was asked to inhale deeply for 5 s after injection of contrast agent, with the glottis tightly closed, and then to perform forced exhalation for 10 s; and (3) a provoked state with sm‐VM, in which the patient was asked to blow the 10‐mL syringe after 5 s of contrast injection until the plunger moved for 10 s (Figure 1). The patients were coached to perform the c‐VM and sm‐VM before the formal examination. There were at least 5 min between the cTCD examinations with different conditions.

The presence and severity of RLS were rated per the categorization system proposed by Jauss and Zanette, 2000 based on the findings of MBs in cTCD examinations for MCA (Table 1). Grade I on cTCD indicates no RLS, whereas grade III (curtain‐like) indicates large RLS.

**TABLE 1:** Severity of right‐to‐left shunt (RLS) based on a four‐level categorization based on the numbers of microbubbles (MBs).

Grade	Number of embolic tracks
Negative	0 MBs
I	1–10 MBs
II	>10 MBs, and no curtain
III	Curtain

*Note*: Patients allocated to grade III were considered to have large RLS.

*Source*: Adapted from Jauss M, Zanette E. Detection of the right‐to‐left shunt with ultrasound contrast agent and transcranial Doppler sonography. Cerebrovascular Diseases, 2000, 10(6), 490–496.

### 2.3 Statistical analyses

Statistical analysis was performed using SPSS 25.0. The chi‐square test was used to evaluate the difference in the positive rate of RLS in cTCD with different conditions. Data were presented as numbers (%). The Bowker test was used to compare the distribution of patients with different degrees of RLS between cTCD examined with c‐VM and sm‐VM. Statistical significance was set at *p* < .05, and the two‐sided test was used.

## 3 RESULTS

### 3.1 Characteristics of the included patients

The baseline characteristics of the included patients who underwent the cTCD examination are shown in Table 2. Overall, 389 patients with clinical suspicion of RLS who underwent cTCD were included in this study. The mean age of the patients was 49.37 ± 14.87 years, and 203 (52.2%) were male. Comorbidities of hypertension, diabetes, and coronary artery disease were presented in 98 (25.2%), 81 (20.8%), and 61 (15.7%) patients, respectively, and 130 (33.4%) of the patients had no known previous diseases. The clinical manifestations included headache in 164 (42.2%) patients, dizziness in 65 (16.7%) patients, diagnosis of transient ischemic attack in 46 (11.8%) patients, and diagnosis of stroke in 35 (9.0%) patients. Among the included patients, 59 (15.2%) were asymptomatic. All the included patients finished the cTCD examinations with three different conditions without any adverse events.

**TABLE 2:** Characteristics of the included patients who underwent the contrast‐enhanced transcranial Doppler (cTCD) examination.

Clinical characteristics	
Age (mean ± SD)	49.37 ± 14.87
Male sex, *N* (%)	203 (52.2%)
Hypertension, *N* (%)	98 (25.2%)
DM, *N* (%)	81 (20.8%)
CAD, *N* (%)	61 (15.7)
Without known diseases, *N* (%)	130 (33.4%)
Clinical manifestations	
Headache, *N* (%)	164 (42.2%)
Dizziness, *N* (%)	65 (16.7)
TIA, *N* (%)	46 (11.8%)
Stroke, *N* (%)	35 (9.0%)
Asymptomatic, *N* (%)	59 (15.2%)

Abbreviations: CAD, coronary artery disease; DM, diabetes mellitus; TIA, transient ischemic attack.

### 3.2 Positive rate of RLS with cTCD detected under different conditions

Of the 389 patients enrolled in the study, 84 (21.6%) were positive for RLS detected with cTCD in a resting state, 133 (34.3%) were positive for RLS in cTCD provoked by c‐VM, and 182 (46.8%) were positive for RLS in cTCD provoked by sm‐VM. The difference in the detection rates for RLS among the three cTCD conditions was statistically significant (*χ* ^2^ = 54.863, *p* < .001, Table 3). Comparisons of the detection rates for RLS between cTCD provoked by sm‐VM and cTCD in resting state and between cTCD provoked by sm‐VM and c‐VM were both statistically significant (both *p* < .001), suggesting that provocation by sm‐VM might further improve the detection rate of RLS as compared to cTCD in resting state and provoked by c‐VM.

**TABLE 3:** Positive rate of right‐to‐left shunt (RLS) with contrast‐enhanced transcranial Doppler (cTCD) detected under resting state and with different provocation methods.

	Patients with RLS, *N* (%)	Patients without RLS, *N* (%)	Pearson's *χ* ^2^	*р*
Resting state	84 (21.6)	305 (78.4)	54.863	<.001
c‐VM	133 (34.2)	256 (65.8)		
sm‐VM	182 (46.8)	207 (53.2)		

Abbreviations: c‐VM, conventional Valsalva maneuver; sm‐VM, syringe‐modified Valsalva maneuver.

### 3.3 Grade of RLS with cTCD detected under different conditions

**FIGURE 2:**
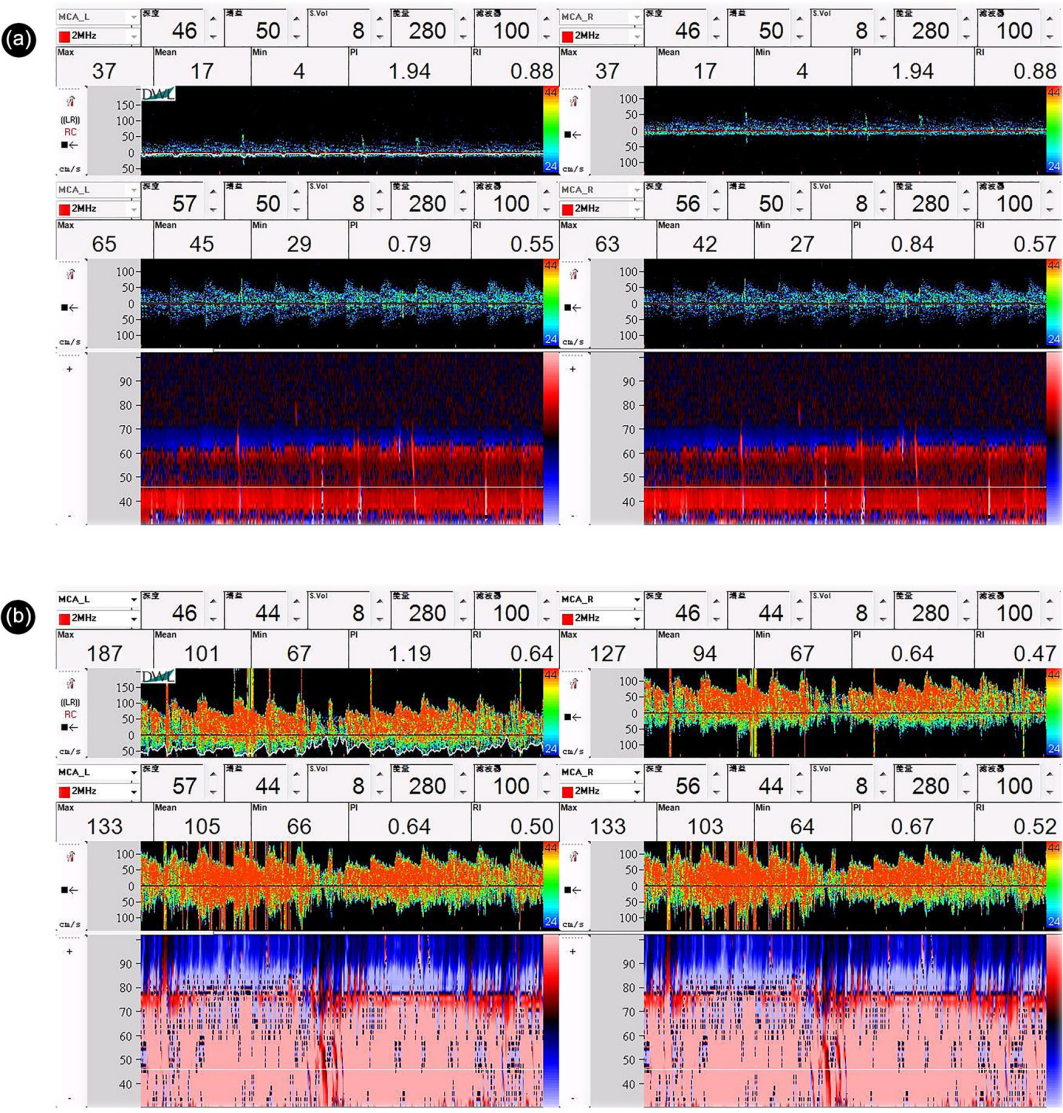
Representative images of the contrast‐enhanced transcranial Doppler (cTCD) examinations in a patient with the conventional and the syringe‐modified Valsalva maneuver: (A) 1 MB was detected (Grade I) during the cTCD examination with the conventional Valsalva maneuver; and (B) A mount of microbubbles (MBs) were detected during the cTCD examination with the syringe‐modified Valsalva maneuver (Curtain, Grade III).

**FIGURE 3:**
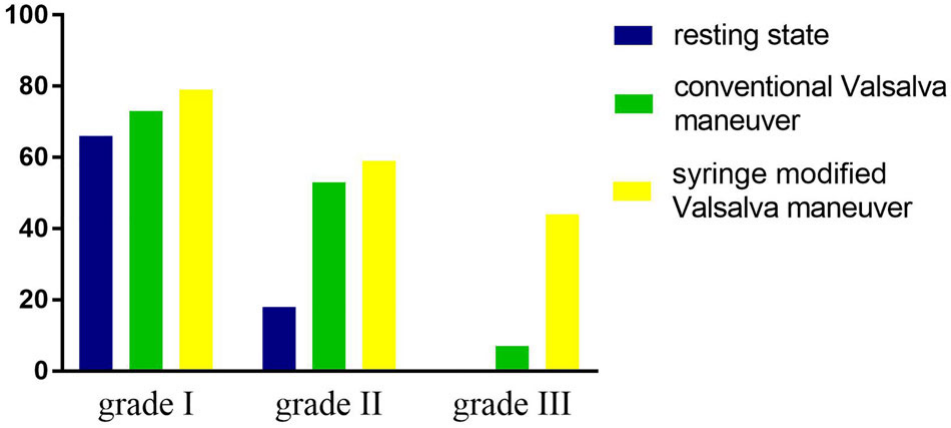
Distribution of patients with different grades of right‐to‐left shunt (RLS) according to the contrast‐enhanced transcranial Doppler (cTCD) examinations detected under resting state and with different provocation methods.

In some typical cases, the cTCD findings on RLS became more remarkable in examination provoked by sm‐VM than in resting state or c‐VM. Figure 2 presents the representative images of the cTCD examinations in patients with c‐VM (grade I, 1 MB) and sm‐VM (grade III, curtain‐like). The distributions of patients with different grades of RLS detected by cTCD under different conditions are shown in Figure 3. During examination in resting state, 66 (17.0%), 18 (4.6%), and 0 (0.0%) patients were classified as grade I, II, and III RLS, respectively, while the numbers for cTCD provoked with c‐VM were 73 (18.8%), 53 (13.6%), and 7 (1.8%), and the numbers for cTCD provoked with sm‐VM were 79 (20.3%), 59 (15.3%), and 44 (11.3%). Comparisons between the distribution of patients with different RLS grades in cTCD examinations provoked with c‐VM and sm‐VM showed significant differences according to the results of the Bowker's test (*p* < .001, Table 4). These findings also suggested that cTCD provoked with sm‐VM was associated with a significantly improved detection rate than TCD provoked with c‐VM.

**TABLE 4:** Distribution of patients according to the grade of right‐to‐left shunt (RLS) with contrast‐enhanced transcranial Doppler (cTCD) detected under resting state and with different provocation methods.

c‐VM (*N*)	sm‐VM (*N*)				
	III	II	I	Negative	Total
III	5	2	0	0	7
II	25	28	0	0	53
I	7	22	40	4	73
Negative	7	7	39	203	256
Total	44	59	79	207	389

Abbreviations: c‐VM, conventional Valsalva maneuver; sm‐VM, syringe‐modified Valsalva maneuver.

## 4 DISCUSSION

In this pilot single‐center comparative study, we evaluated and compared the detection rate of RLS via cTCD examinations under three different conditions. The results showed that compared to cTCD examinations under resting state or with the provocation of c‐VM, cTCD investigation with a newly proposed sm‐VM was associated with a significantly improved detection rate for overall patients with RLS. Besides, cTCD detected with sm‐VM was also associated with a higher proportion of patients with grade III RLS than those detected at resting state and with c‐VM. All included patients finished the cTCD examinations and reported no adverse events related to the investigation. These findings suggest that compared to cTCD detected at resting state and with c‐VM, cTCD with sm‐VM could further increase the positive detection rate of RLS, which may be an already feasible screening strategy for RLS in a high‐risk population.

Our results showed that c‐VM provoked cTCD was associated with a higher RLS detection rate than cTCD examination at resting state, which was consistent with the findings of the previous studies (Gonzalez‐Alujas et al., 2011; Van et al., 2010). Clinically, RLS could be classified as intrinsic shunt and latent shunt. Although most of the intrinsic RLS could be detected via cTCD at a resting state, latent RLS may only be detected via cTCD when the intrathoracic pressure increases, such as during coughing and performing the c‐VM. A previous study showed that the right atrial pressure (RAP) was 6.6 ± 2.6 mmHg at rest, which could be increased to 21.6 ± 11.9 mmHg when the c‐VM was performed (Van et al., 2010). Therefore, it could be speculated that performing the c‐VM may increase the detection rate of RLS by increasing the RAP to a level higher than the left atrial pressure, making the latent shunt intrinsic. This may also explain the low detection rate of cTEE compared to cTCD for RLS in previous studies (Gonzalez‐Alujas et al., 2011). Anesthesia is generally needed for cTEE, which makes performing c‐VM inadequate or impossible compared to the detection via cTCD.

The most important finding of our study was that the novel proposed sm‐VM‐provoked cTCD could further increase the detection rate of RLS compared to cTCD via c‐VM provocation. A previous study including 38 patients undergoing PFO closure showed that in detecting RLS, cTCD with immediate feedback through forced expiration against a manometer to 40 mmHg was more sensitive than echocardiography (Van et al., 2010). Consistently, another study showed that blowing air into a 10 mL syringe to move the plunger could generate an intrathoracic pressure of 40 mmHg, thus achieving the recommended intrathoracic pressure for optimal VM performance (Smith & Boyle, 2009). In our study, moving the plunger by blowing the syringe is likely equivalent to controlling the VM of 40 mmHg with a pressure gauge, which may be the essential underlying mechanism for the improved detection rate of RLS with the sm‐VM‐provoked cTCD. Accordingly, the detection rate of RLS in our study with sm‐VM‐provoked cTCD was similar to that of the previous study with cTCD using a pressure gauge controlled VM to 40 mmHg method (46.8% vs. 47.3%) (Guo et al., 2016). In the previous study by Van et al., c‐VM only increased the RAP to 21.6 ± 11.9 mmHg, which may further explain the lower detection rate or RLS in cTCD using c‐VM as compared to that of sm‐VM‐provoked cTCD.

Clinically, it has been recommended that pressure gauge‐controlled VM could be used to reinforce the effect of VM on intrathoracic pressure changes (Guo et al., 2016; Van et al., 2010). Generally, patients were requested to blow forcefully into the tube connected to the pressure gauge to make the mercury of the pressure gauge reach 40 mmHg and maintain it for 10 s to increase the detection rate of RLS with cTCD. However, the pressure gauges used are mainly mercury sphygmomanometers, which may expose the staff and patients to mercury leakage and contamination risk. Moreover, there may also be a cross‐transmission risk if the rubber tube connected to the sphygmomanometer is reused. In only study, blowing with a 10 mL syringe was used, and the efficacy of which has been approved to be equal to the effective VM, which increases the intrathoracic pressure to 40 mmHg (Smith & Boyle, 2009). The method is easy to understand and perform. The target action could be clearly described to the patient and easily achieved during the examination. Besides, cardiologists have widely and effectively used similar protocols in treating hemodynamically stable supraventricular tachycardia with approved safety (Appelboam et al., 2015; Kotruchin et al., 2022; Mehta et al., 1988). No expensive instrument but a disposable 10‐mL syringe is used, which is also cost‐effective. All of the above advantages make the sm‐VM a feasible and potentially effective strategy for improving the detection rate of RLS via the cTCD examination.

The study has certain limitations. First, this is a single‐center study with a limited sample size. The study's results should be validated by large‐scale clinical investigations in the future. Second, no comparison was performed regarding the detection rate of sm‐VM‐provoked cTCD and cTEE. Future studies are also warranted. Finally, the sensitivity and specificity of sm‐VM‐provoked cTCD for detecting RLS should be analyzed in the future.

## 5 CONCLUSION

In conclusion, the study results indicate that compared to cTCD detected at resting state and with c‐VM, cTCD with sm‐VM could further increase the positive detection rate of RLS. The proposed cTCD with sm‐VM is effective, safe, and inexpensive, which may become an already feasible method for screening RLS in high‐risk populations.

## AUTHOR CONTRIBUTIONS

Jinfeng Deng participated in drafting the work, acquisition, analysis, and interpretation of data. Yan Luo performed the cTCD examinations. Zhijian Luo and Hongrui Zhan participated in the data acquisition and interpretation. Feng Zhou participated in the conception and design of the work. Songbiao Li conceived the study, participated in its design and coordination, and helped draft the manuscript. All authors read and approved the final manuscript.

## CONFLICT OF INTEREST STATEMENT

We declare that we have no conflicts of interest.

## FUNDING INFORMATION

2022 Zhuhai Social Development Science and Technology Plan Medical and Health Project, Project Number: 2220004000274

## CONSENT FOR PUBLICATION

All data published here are under the consent for publication.

### PEER REVIEW

The peer review history for this article is available at https://publons.com/publon/10.1002/brb3.3304.

## Data Availability

The datasets generated and analyzed during the present study are available from the corresponding author upon reasonable request.
